# Identification of an immune prognostic 11-gene signature for lung adenocarcinoma

**DOI:** 10.7717/peerj.10749

**Published:** 2021-01-20

**Authors:** Tao Yang, Lizheng Hao, Renyun Cui, Huanyu Liu, Jian Chen, Jiongjun An, Shuo Qi, Zhong Li

**Affiliations:** 1 Department of Hematology and Oncology, Dongzhimen Hospital, the First Clinical Medical College of Beijing University of Chinese Medicine, Beijing, China; 2 Department of Thyroid, Dongzhimen Hospital, the First Clinical Medical College of Beijing University of Chinese Medicine, Beijing, China

**Keywords:** LUAD, TME, Prognosis, Gene signature

## Abstract

**Background:**

The immunological tumour microenvironment (TME) has occupied a very important position in the beginning and progression of non-small cell lung cancer (NSCLC). Prognosis of lung adenocarcinoma (LUAD) remains poor for the local progression and widely metastases at the time of clinical diagnosis. Our objective is to identify a potential signature model to improve prognosis of LUAD.

**Methods:**

With the aim to identify a novel immune prognostic signature associated with overall survival (OS), we analysed LUADs extracted from The Cancer Genome Atlas (TCGA). Immune scores and stromal scores of TCGA-LUAD were downloaded from Estimation of STromal and Immune cells in MAlignant Tumour tissues Expression using data (ESTIMATE). LASSO COX regression was applied to build the prediction model. Then, the prognostic gene signature was validated in the GSE68465 dataset.

**Results:**

The data from TCGA datasets showed patients in stage I and stage II had higher stromal scores than patients in stage IV (*P* < 0.05), and for immune score patients in stage I were higher than patients in stage III and stage IV (*P* < 0.05). The improved overall survivals were observed in high stromal score and immune score groups. Patients in the high-risk group exhibited the inferior OS (*P* = 2.501*e* − 05). By validating the 397 LUAD patients from GSE68465, we observed a better OS in the low-risk group compared to the high-risk group, which is consistent with the results from the TCGA cohort. Nomogram results showed that practical and predicted survival coincided very well, especially for 3-year survival.

**Conclusion:**

We obtained an 11 immune score related gene signature model as an independent element to effectively classify LUADs into different risk groups, which might provide a support for precision treatments. Moreover, immune score may play a potential valuable sole for estimating OS in LUADs.

## Introduction

Lung cancer is the leading cause of cancer related mortality in the US, especially among men aged ≥40 years and women aged ≥60 years, and will lead to an estimated 135,720 deaths in 2020 (Siegel, Miller & Jemal, 2020). LUAD, which is a main pathological subtype of lung cancer, accounts for approximately 50% lung cancer cases with the survival rate of 4–17%. With tumour genetic testing having been a significant clue to the treatment, the LUADs who have *EGFR* mutations and *ALK* or *ROS1* fusions will be sensitivity to kinase inhibitors (Datta et al., 2019; Ly, Olin & Smith, 2018; Ruiz-Patino et al., 2018; Yoda et al., 2018). For the majority of LUADs, however, the prognosis remains poor for local progression or metastases at the time of diagnosis (Herbst, Morgensztern & Boshoff, 2018).

The views of cancer have shifted over the last 10 years from an autonomous cellular disease to the interactive system between cancer cells and TME (Gajewski, Schreiber & Fu, 2013; Gentles et al., 2015). TME, which was first proposed in the 1970s (Verloes & Kanarek, 1976), is governed by a complex network of biological pathways and is consisted of endothelial cells, mesenchymal cells, immune cells, inflammatory mediators and extracellular matrix molecules (Hanahan & Coussens, 2012). In recent years, TME has drawn more attention due to its importance in the initiation and progression of lung cancer (Altorki et al., 2018; Barker et al., 2015), which may become even more dominant in the future. Further features of the tumour molecular landscape have the potential to identify molecular targets and to act as novel biomarkers that impact disease progression (Jamal-Hanjani et al., 2017). In addition, evidence has shown that the identification of molecular biomarkers can provide prognostic value for LUADs (Wang et al., 2019; Zhang, Zhang & Yu, 2019). For example, high *ARL4C* expression is an essential prognostic factor in LUAD (Kimura et al., 2020). However, a single gene biomarker is insufficient to produce comprehensive predictive effects and immune prognostic models were proposed for LUADs recently (Luo et al., 2020; Yang et al., 2019; Yue, Ma & Zhou, 2019). Until now, only few biomarkers for LUADs have been developed and there is a need to identify more pathways for new biomarkers including sensitive biomarkers which can provide individual therapeutic strategies for patients. In this work, using both ESTIMATE algorithm-derived immune scores and TCGA database of LUAD cohorts, we extracted a list of TME associated genes named CD70, CXorf21, CD74, RUBCNL, BIRC3, TESC, STAP1, INSL4, OBP2A, HLA-DOB and CIITA. Besides, a risk score model was constructed based on 11-gene signature, which may help to improve the prognosis prediction of LUAD.

## Materials & Methods

### Data download and processing

The mRNA FPKM data and clinical data of LUAD were obtained from TCGA-LUAD project (Date: 2020-05-02, https://portal.gdc.cancer.gov/). Immune scores and stromal scores of TCGA-LUAD were downloaded from ESTIMATE database (Date: 2020-05-02, https://bioinformatics.mdanderson.org/estimate/). The GSE68465 dataset was obtained from Gene Expression Omnibus (GEO, Date: 2020-05-08, https://www.ncbi.nlm.nih.gov/geo/). The TCGA samples’ clinical information, mRNA FPKM data and immune-related scores were collected and integrated for the next step. The eligible samples were screened by following criteria: (a) samples identified as lung adenocarcinoma by TCGA-ID, (b) samples with complete clinical information, including gender, age, TNM stage and AJCC stage, (c) samples can be matched with the corresponding immune score information from the ESTIMATE database. In addition, all the analyses in this study were conducted by R software (version 3.6.1).

### Grouped by ESTIAMTE score and OS analysis

Based on median values, all samples were categorized into two groups, namely high immune/stromal score group and low immune/stromal score group. Kaplan Meier analysis was used to analyze the OS between high and low score groups, and survival curves were conducted by *R package survival* (Terry & Patricia, 2000). Statistical significance was confirmed at *P*-value < 0.05.

### Identification of DEGs

According to the results of OS analysis, we selected DEGs between high and low groups which are closely related to OS. DEGs analysis was performed using *R package limma* (Ritchie et al., 2015). —log_2_ Fold change— > 1 and adj. *P*-value < 0.05 were set as the cutoffs to screen for DEGs. All DEGS between high and low immune score groups were regarded as the immunological TME related genes of LUAD.

### GO enrichment analysis of DEGs

*ClusterProfiler* is an *R package* for the process of biological-term classification and the enrichment analysis of gene clusters (Yu et al., 2012). We used *ClusterProfiler* to analyze the function of the immunological TME associated genes of LUAD. GO bioinformatics tool was used to annotate genes and analyze genes by molecular function, biological process and cellular component. Statistical significance was considered at *P*-value < 0.05.

### Construction of prognosis model

Univariate COX regression and LASSO regression analysis were used in the construction of prognostic model. Firstly, we used univariate COX regression analysis to filter the chosen genes that have a significant impact on OS of TCGA-LUAD cohorts. Then, genes with *P*-value < 0.05 were filtered by LASSO regression analysis. L1-normalization can penalize the weight of the parameters and achieve dimension reduction, which is widely used in LASSO regression analysis. This process was achieved by the *glmnet* package for R (Friedman, Hastie & Tibshirani, 2010). Risk scores of LUADs were computed via using the following formula: risk score = ${\mathop{\sum }\nolimits }_{j=1}^{n}\beta j\times Xj$, where *β*_j_ represents the coefficient and X_j_ indicates the relative expression levels of each selected gene. Risk scores were estimated by involving these selected genes, and the median risk score was chosen as a cutoff value to separate TCGA-LUAD into high and low risk groups. Then we validated those prognostic gene signature in the GSE68465 dataset, and the same computing method was used to score the patients in GEO datasets as in the training set. In addition, we have conducted univariate COX analysis and multi-variable COX analysis separately to test the prognostic ability of clinical features including risk score. ROC (receiver operating characteristic) curve is an important tool to evaluate model performance, which is already applied in biomedical field (Le et al., 2019; Le, Yapp & Yeh, 2019). Therefore, we chosed The ROC curves to further evaluate the predictive ability of the model we got.

### Construction of co-expression network and hub gene analysis

Based on the above analysis, we obtained the TME related genes which have prognostic potential. Then, we used online database cBioportal (http://www.cbioportal.org/) to get co-expression genes, and the most 20 significant genes were filtered by *P*-value. The co-expression network was visualized by Cytoscape, and the hub genes of network were selected by MCC score calculated by cytoHubba, which is a network topology analysis tool of Cytoscape. Furthermore, we conducted the immune infiltration analysis in TIMER database (https://cistrome.shinyapps.io/timer/). Scatter plots were used to display correlation between hub genes and immune cells in LUAD cohort.

### Development of nomogram

In the TCGA-LUAD cohorts, age (<=65 or >65), gender (male or female), AJCC stage (stage I–II or stage III–IV), T stage (T 1-2 or T 3-4), N stage (N+ or N0), M stage (M1 or M0) and risk score (high or low score) were used to construct a nomogram. Calibration curves were plotted to evaluate the consistency between actual and predicted survival. Furthermore, the concordance index (C-index) was calculated to assess the model performance for predicting prognosis. Generally, C-index ranging from 0.50 to 0.70, 0.71 to 0.9 and 0.91 to 1.0 indicate the low, medium and high accuracy of model, respectively. All above analysis processes were involved with the R package *rms* (Frank, 2020).

## Results

**Figure 1:**
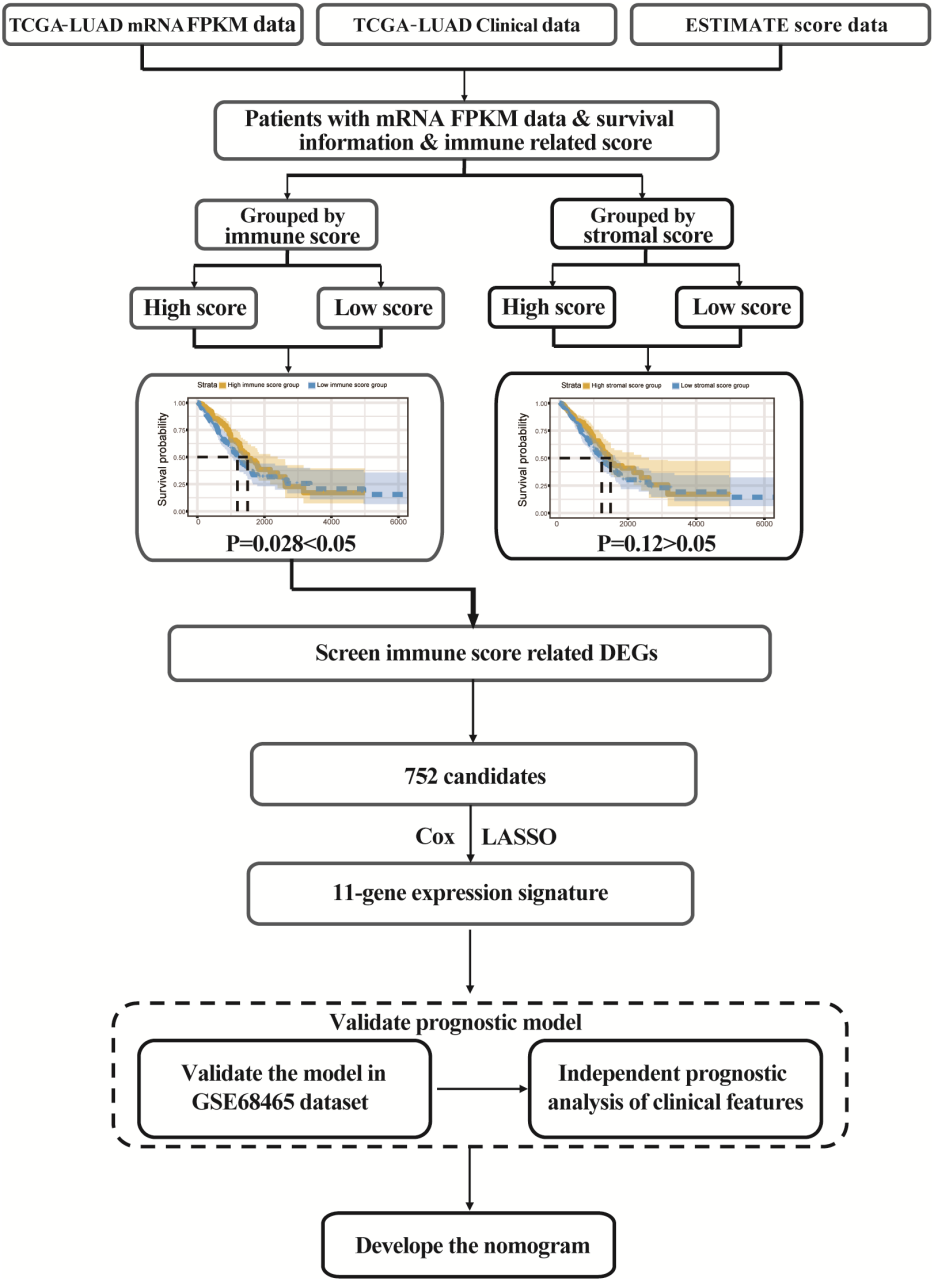
Study flowgraph. This is the study flowgraph of this work.

The flowgraph of this study is shown in Fig. 1. The TCGA LUAD mRNA FPKM data and clinical data were integrated with the score, and 477 candidate patients were selected. The immunological TME-related signatures of LUAD were determined by univariate COX regression and LASSO regression analysis, and the prognostic model was established.

### ESTIMATE scores are associated with TCGA-LUAD clinical features

**Figure 2:**
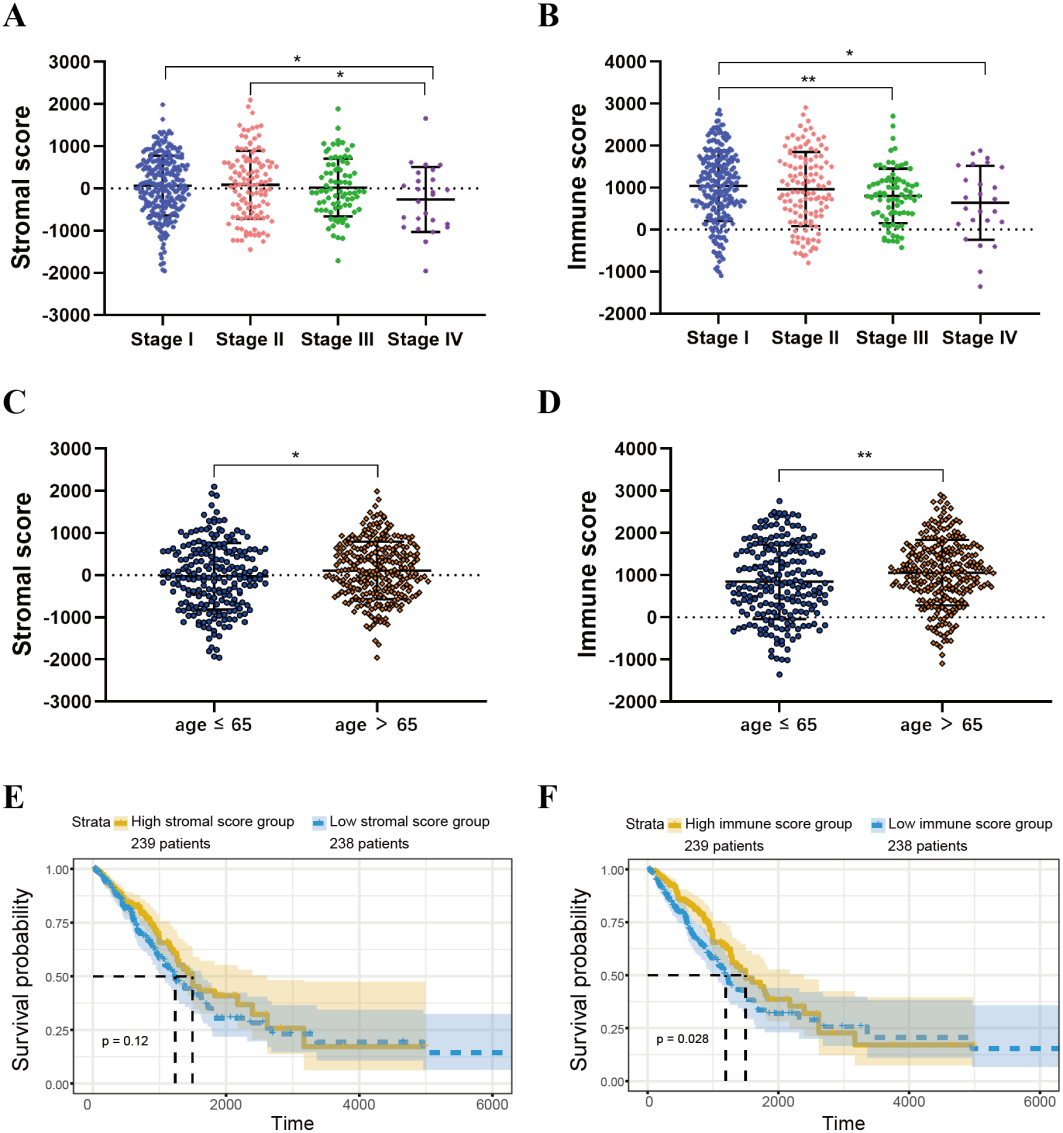
Immune scores and stromal scores are associated with LUAD clinical features and their overall survival. (A) Distribution of stromal scores of different AJCC stages. (B) Distribution of immune scores of different AJCC stages. (C) Distribution of stromal scores for high- and low-age cases. (D) Distribution of immune scores for high- and low-age cases. (E) LUAD cases were divided into two groups based on their median stromal score. As shown in the Kaplan–Meier survival curve, median survival of the high score group is longer than low score group; however, it is not statistically different as indicated by the log-rank test *p* = 0.12. (F) LUAD cases were also divided into two groups based on their median immune score. The median survival of the high score group is longer than the low score group, as indicated by the log-rank test, *p* = 0.028.

ESTIMATE algorithm could be used to calculate immune and stromal scores to predict the infiltration of non-tumour cells in TME (Yoshihara et al., 2013). We obtained the stromal scores and immune scores of TCGA-LUAD samples from ESTIMATE database. Based on different clinical stages, it showed that stage II patients have the highest stromal score where the lowest stromal score is achieved by patients in stage IV. The stromal scores of stage I and stage II patients are obviously higher than patients in stage IV (*P* < 0.05) (Fig. 2A). For immune score, stage I patients are significantly higher than patients in stage III and stage IV (*P* < 0.05) (Fig. 2B). In addition, we separated all samples into two groups, including age ≤ 65 group and age >65 group. The stromal score and immune score of samples in age >65 group are notably higher than samples in age ≤ 65 group (*P* < 0.05) (Figs. 2C–2D).

### Identification and selection of the immunological TME related DEGs and GO analysis

**Figure 3:**
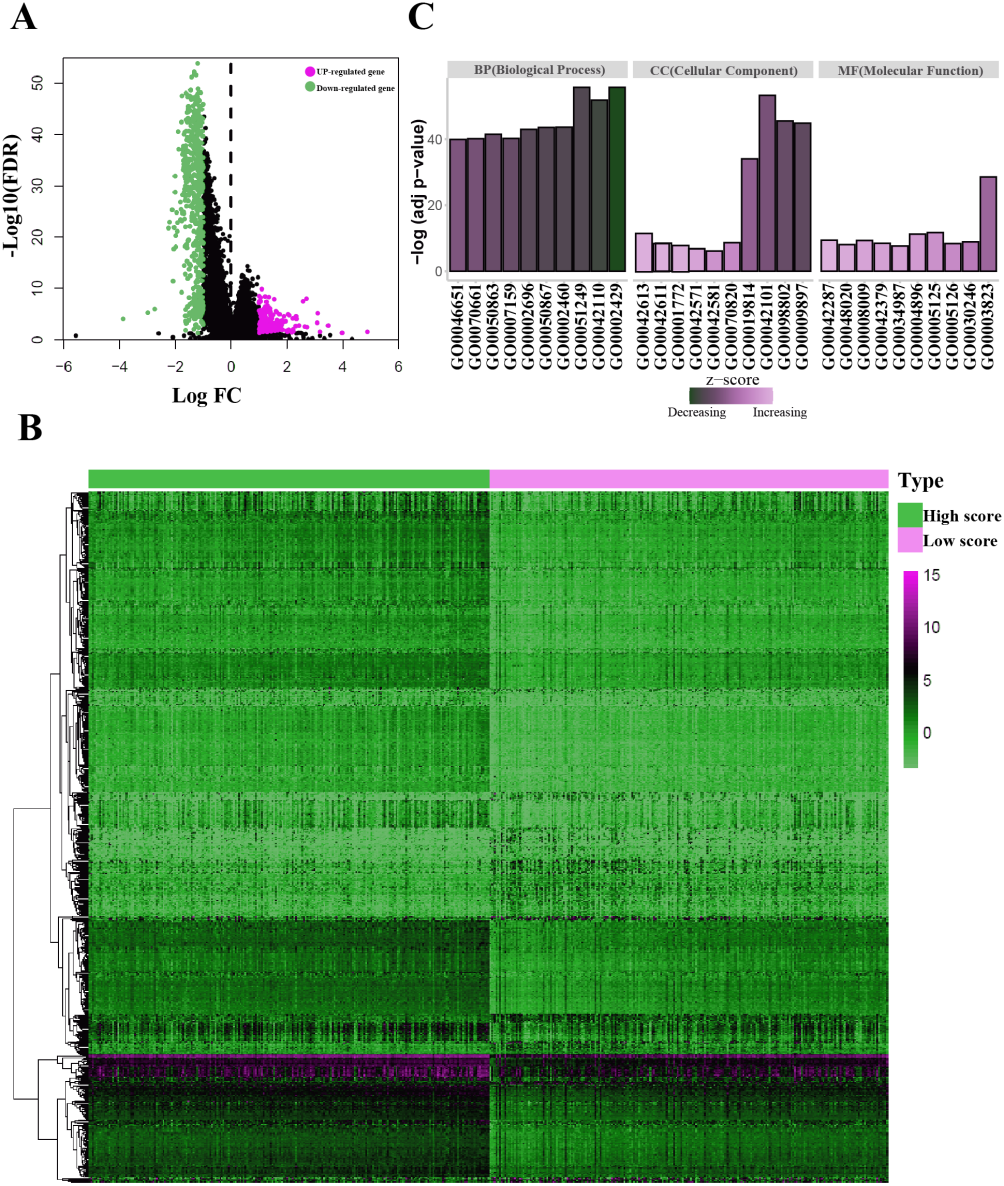
Identification of the immunological TME related DEGs. (A) Volcano plots showing DEGs in immune score groups, it includes 144 up-regulated genes and 608 down-regulated genes. (B) Heatmap of the DEGs of immune scores of high score group vs. low score group. (adj. *p* < 0.05, fold change >2). (C) GO enrichment analysis were conducted by clusterprofiler, the bar-plot shows the ten most significant terms of MF, BP and CC.

For both immune and stromal score, all patients were divided into high-score groups and low-score groups, respectively. Firstly, we conducted the OS analyses between high-score and low-score groups in two ways. In stromal score-related groups, the OS of high-score group was longer than the low-score group (median survival time 1492 days vs 1235 days), but there was non-statistically significant advantage (*P* = 0.12) (Fig. 2E). As for immune score-related groups, the median survival time were 1499 days in high-score group and 1194 days in low-score group, there was statistically significant advantage in overall survival (*P* = 0.028) (Fig. 2F). Furthermore, DEGs were selected in immune score-related groups by *R package limma*. For immune score-related groups, we filtered 752 DEGs including 144 up-regulated genes and 608 down-regulated genes, which were regarded as the immunological TME related genes of LUAD (—log_2_ Fold change— > 1, adj.p < 0.05) (Figs. 3A–3B). Then, GO analysis was performed and results indicated that alterations in molecular function (MF) of these genes have been significantly enriched in *antigen binding, cytokine activity, cytokine receptor activity, MHC protein binding and chemokine activity* et al. (Fig. 3A). The immunological TME related DEGs participate in biological processes (BP) including immune response-activating cell surface receptor signaling pathway, regulation of lymphocyte activation, T cell activation, adaptive immune response based on somatic recombination of immune receptors built from immunoglobulin superfamily domains, and positive regulation of cell activation et al. (Fig. 3B). In addition, the DEGs were mainly enriched in *T cell receptor complex, plasma membrane receptor complex, external side of plasma membrane, immunoglobulin complex and MHC class II protein complex* et al. which changed in cellular component (CC) (Fig. 3C, Table 1).

**Table 1:** Top 10 terms of BP, MF, CC.

Category	GO-ID	GO-Term	Adj *P* value
MF	GO0003823	Antigen binding	2.82E−29
MF	GO0005125	Cytokine activity	1.98E−12
MF	GO0004896	Cytokine receptor activity	5.95E−12
MF	GO0042287	MHC protein binding	3.85E−10
MF	GO0008009	Chemokine activity	4.74E−10
MF	GO0030246	Carbohydrate binding	1.33E−09
MF	GO0042379	Chemokine receptor binding	3.36E−09
MF	GO0005126	Cytokine receptor binding	4.14E−09
MF	GO0048020	CCR chemokine receptor binding	8.63E−09
MF	GO0034987	Immunoglobulin receptor binding	2.28E−08
BP	GO0002429	Immune response-activating cell surface receptor signaling pathway	1.94E−56
BP	GO0051249	Regulation of lymphocyte activation	1.94E−56
BP	GO0042110	T cell activation	1.58E−52
BP	GO0002460	Adaptive immune response based on somatic recombination of immune receptors built from immunoglobulin superfamily domains	2.32E−44
BP	GO0050867	Positive regulation of cell activation	2.7E−44
BP	GO0002696	Positive regulation of leukocyte activation	1.07E−43
BP	GO0050863	Regulation of T cell activation	3.29E−42
BP	GO0007159	Leukocyte cell–cell adhesion	6.12E−41
BP	GO0070661	Leukocyte proliferation	6.89E−41
BP	GO0046651	Lymphocyte proliferation	1.23E−40
CC	GO0042101	T cell receptor complex	5.35E−54
CC	GO0098802	Plasma membrane receptor complex	3.06E−46
CC	GO0009897	External side of plasma membrane	1.4E−45
CC	GO0019814	Immunoglobulin complex	8.93E−35
CC	GO0042613	MHC class II protein complex	5.75E−12
CC	GO0070820	Tertiary granule	2.3E−09
CC	GO0042611	MHC protein complex	3.26E−09
CC	GO0001772	Immunological synapse	1.82E−08
CC	GO0042571	Immunoglobulin complex, circulating	1.58E−07
CC	GO0042581	Specific granule	7.85E−07

### Construction of prognosis model based on the immunological TME related DEGs

**Figure 4:**
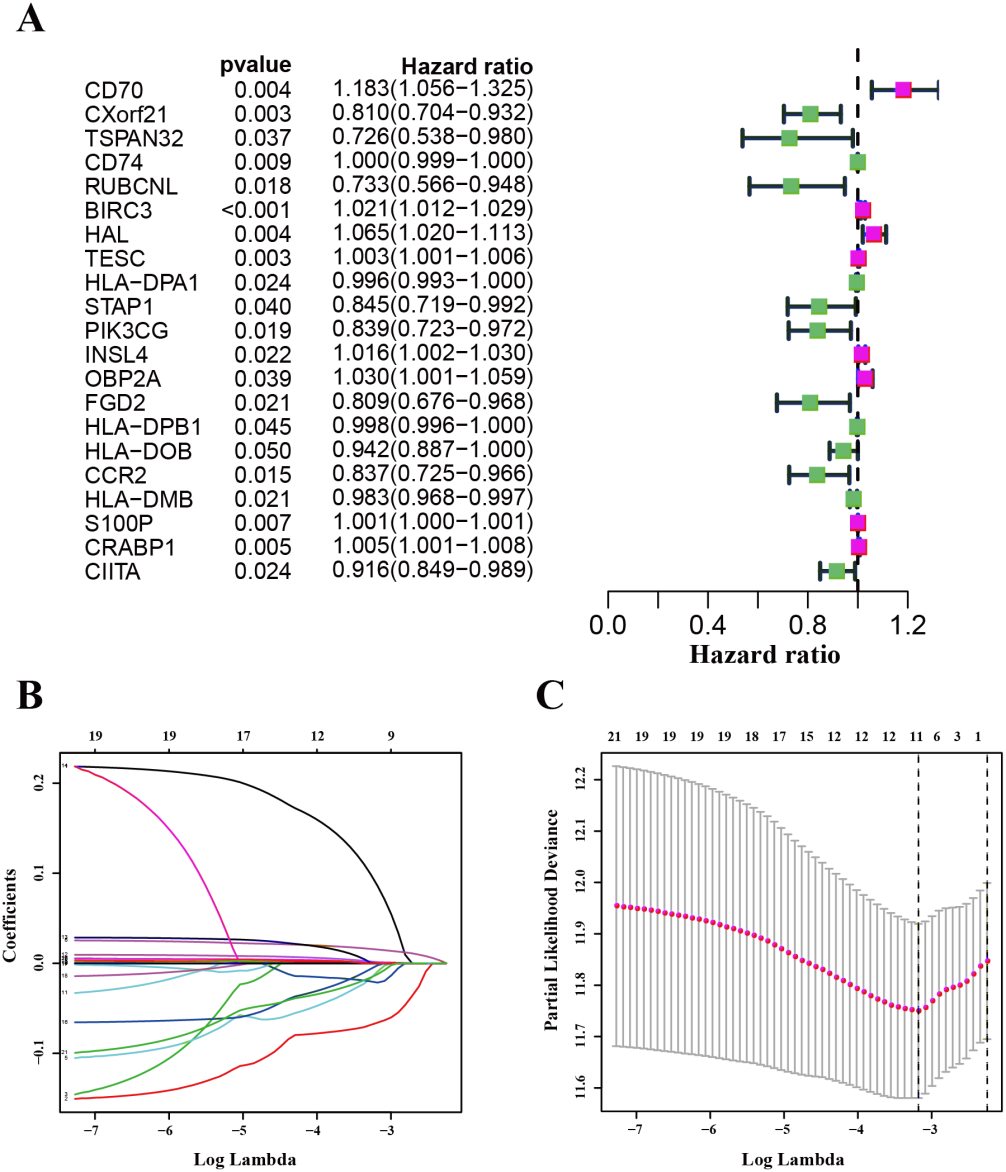
Identification and validation of the prognostic gene signature. (A) The forest map showing 21 genes significantly correlated with overall survival in the univariable COX regression analysis. (B) LASSO coefficient profiles of the 11 genes in TCGA-LUAD. (C) A coefficient profile plot was generated against the log (lambda) sequence. Selection of the optimal parameter (lambda) in the LASSO model for TCGA-LUAD.

**Figure 5:**
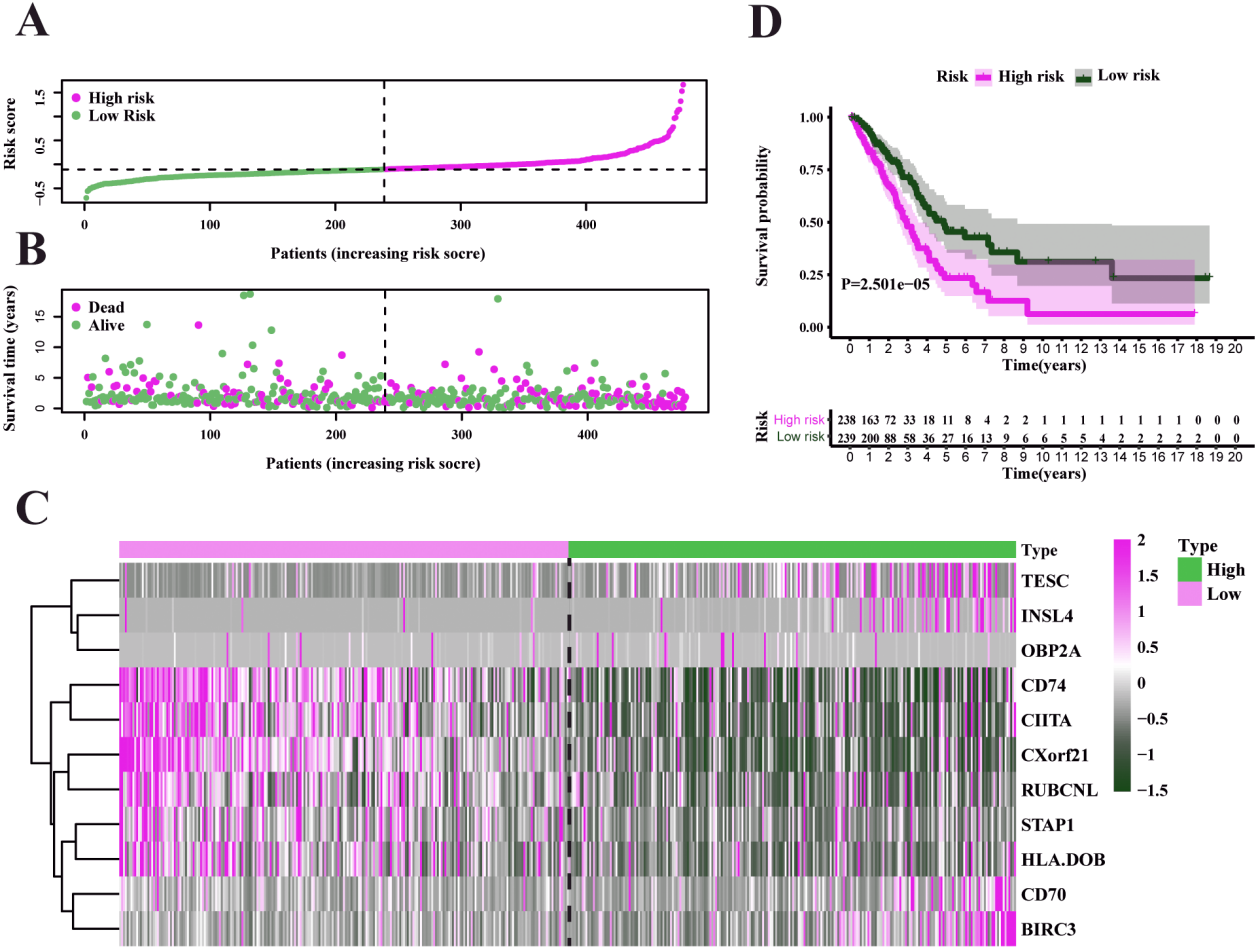
Characteristics of the prognostic gene signature and OS analysis in TCGA. (A) Risk score distribution and survival overview in the TCGA-LUAD set. (B) Heatmap showing the expression profiles of the signature in low- and high-risk groups. (C) Patients in the high-risk group exhibited worse overall survival compared to those in the low-risk group (*P* = 2.501e−05).

We used the TCGA-LUAD cohorts to train the prognosis model. Through univariate COX regression analysis, 21 genes were selected for LASSO regression analysis (Fig. 4A). At last, 11 genes were filtered by LASSO regression analysis (Figs. 4B–4C). Then, we calculated and ranked the risk score for each sample in the TCGA-LUAD set. Thus, the 477 patients in training set were divided into two groups: a low-risk group (*n* = 239) and a high-risk group (*n* = 238), and the median of risk score was regarded as cut-off value (Figs. 5A–5B). Figure 5A also shows the survival overview in the training set. The gene expression profiles in high and low risk LUAD groups were displayed in a heatmap (Fig. 5C). The Kaplan–Meier curve was plotted to display survival situation between high and low risk groups, and log-rank test showed that patients in the low-risk group have significantly better OS compared to those in the high-risk group (median survival time 4.87 years vs 2.94 days, *P* = 2.501*e* − 5 < 0.01) (Fig. 5D).

### Validation and evaluation of the prognosis model

**Figure 6:**
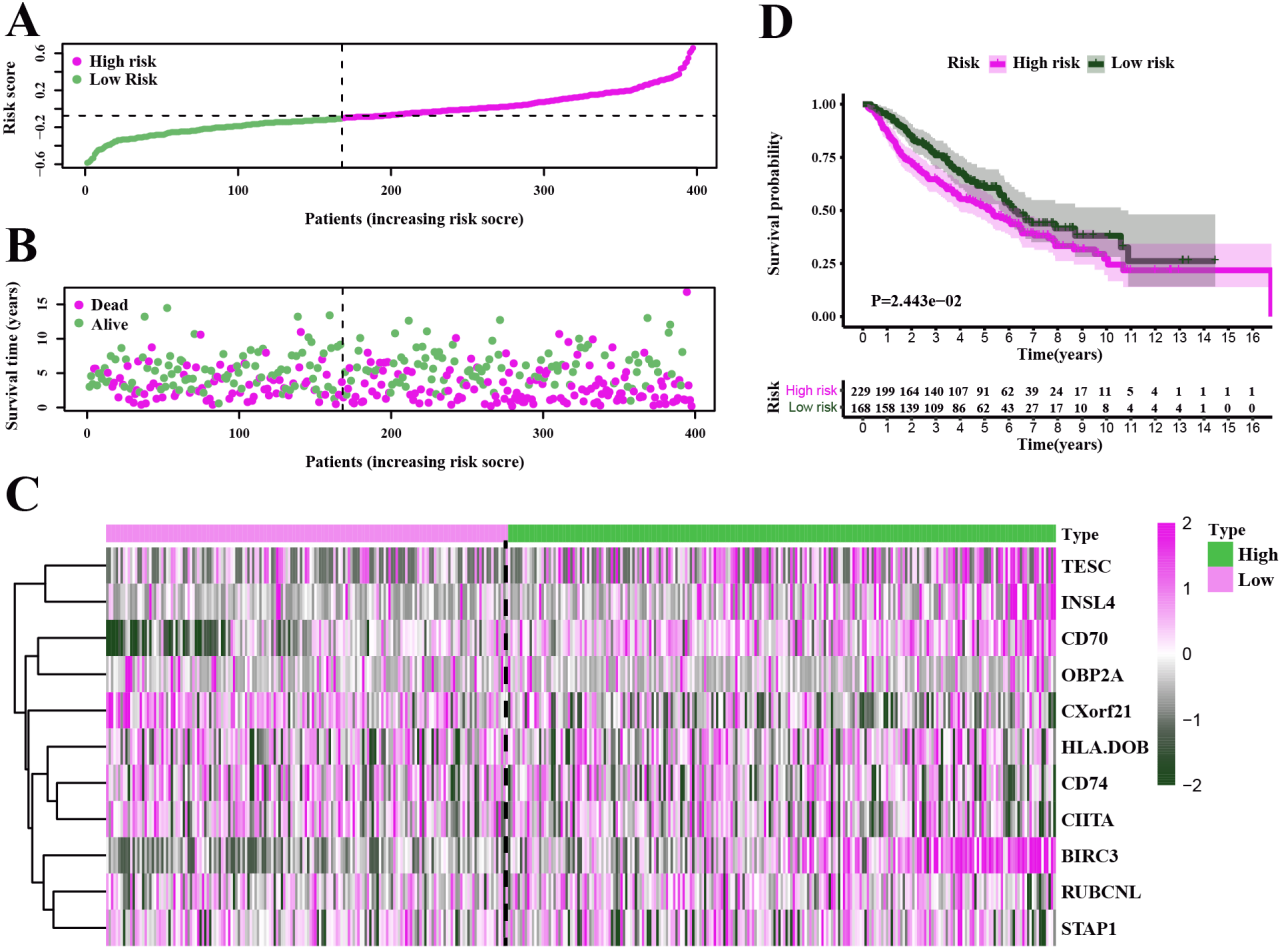
Characteristics of the prognostic gene signature and OS analysis in GSE68465. (A–B) The risk score distribution and the survival overview in the GEO cohort. (C) The gene expression profiles in different risk groups. (D) The survival curve indicated a superior OS in the low-risk group compared to the high-risk group (median survival time 6.22 years vs 5.26 days, *P* = 2.443*e* − 2 < 0.05).

To test our results in the training set, we validated the prognostic performance of 11-gene signature in the GEO dataset (GSE68465). We filtered the 397 LUAD patients with clinical information from GSE68465. The risk score was calculated for each patient in the testing set by adopting the same method in the TCGA set. The risk score distribution and the survival overview in the GEO cohort were showed in Figs. 6A–6B. Based on the median cut-off value, the patients in GSE68465 were also divided into high-risk (*n* = 229) and low-risk (*n* = 168) groups. A heatmap was plotted to display the gene expression profiles in different risk groups (Fig. 6C). The survival curve indicated a superior OS in the low-risk group compared to the high-risk group (median survival time 6.22 years vs 5.26 days, *P* = 2.443*e* − 2 < 0.05) (Fig. 6D).

### Independent prognostic analysis of LUAD

**Figure 7:**
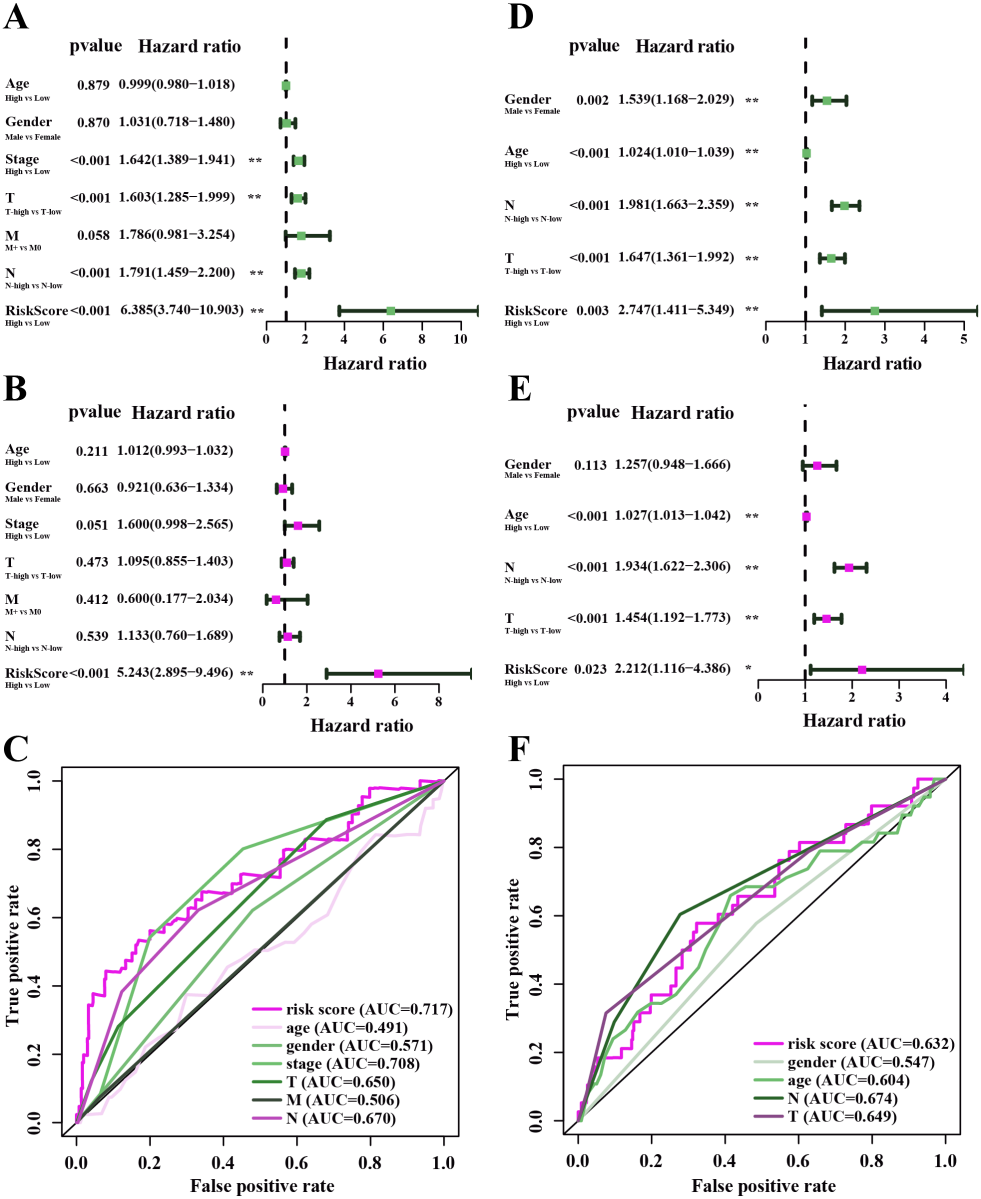
The performance of independent prognostic analysis of risk factors. (A) Univariate Cox regression analysis, (B) multivariate Cox regression analysis. Forest plot of the association between risk factors and survival of TCGA-LUAD. (C) The area under the curve (AUC) was calculated for ROC curves, and sensitivity and specificity were calculated to assess the risk factors performance. (D) Univariate Cox regression analysis, (E) multivariate Cox regression analysis. Forest plot of the association between risk factors and survival of GSE68465. (F) AUC curves were calculated for ROC curves, and sensitivity and specificity were calculated to assess the risk factors performance.

In the TCGA-LUAD cohorts, clinical features including age, gender, AJCC stage, T stage, N stage, M stage and risk score were evaluated in univariable COX and multi-variable COX analysis. From the results of univariable COX analysis, we can see that the AJCC stage, T stage, N stage and risk score were dramatically associated with the OS of TCGA-LUAD (Fig. 7A). In the multi-variable COX analysis, however, only the risk score showed significant associated with the OS of TCGA-LUAD (Fig. 7B). To evaluate the competitive performance of the above clinical features, time-dependent ROC curve analysis was measured, and the highest area under the curve (AUC) score was 0.717, which was contributed by risk score. The risk score demonstrated the satisfactory performance of survival prediction in the TCGA-LUAD. The rank order of AUC scores across TCGA-LUAD from highest to lowest is risk score, AJCC stage, N stage, T stage, N stage, gender, M stage and age (Fig. 7C). In the GSE68465 dataset, clinical features are consisted of gender, age, T stage, N stage and risk score. Univariable COX and multi-variable COX analysis were conducted as well. The results of univariable COX analysis showed that all clinical features were remarkably associated with the OS of GSE68465 cohorts (Fig. 7D), and age, T stage, N stage and risk score showed significantly association with the OS of GSE68465 cohorts in the multi-variable COX analysis (Fig. 7E). ROC curve analysis was also measured; the rank order of AUC scores across GSE68465 cohorts from the highest to the lowest is N stage, T stage, risk score, age and gender, and the AUC value for the risk score was 0.632 (Fig. 7F).

### Co-expression network of genes in the model and TIMER analysis

**Figure 8:**
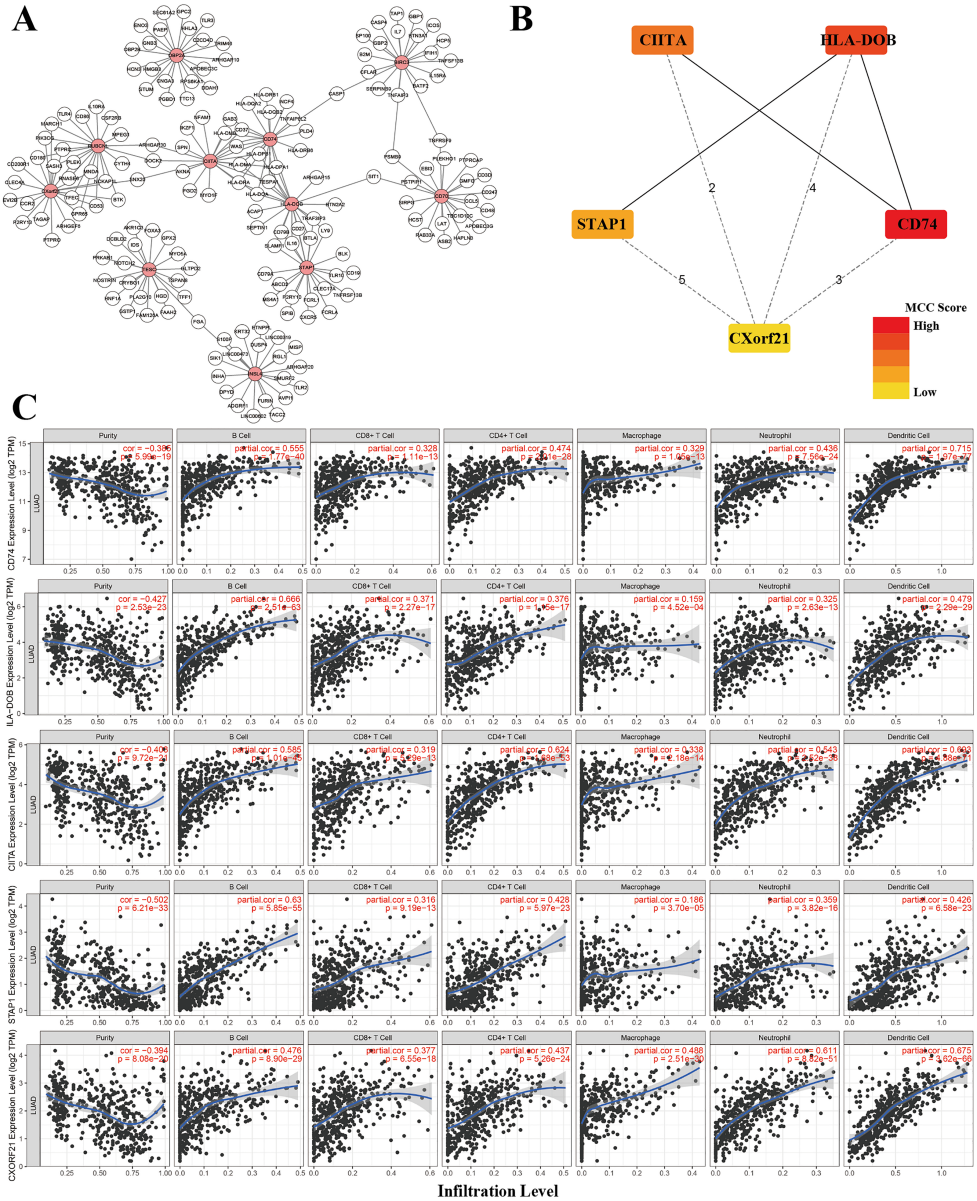
Co-expression network of genes in the model and TIMER analysis. (A) Co-expression network was constructed by online network tool cBiopotal. (B) The five hub genes (CD74, HLA-DOB, CIITA, STAP1 and CXorf21). (C) Spearman’s correlation evaluated associations between gene and immune cells.

Under the univariate COX regression and LASSO regression analysis, 11 genes were incorporated into the model. The co-expression network was constructed by online network tool cBiopotal (Fig. 8A), and the five most important genes were selected by cytoHubba, which is a plug-in of Cytoscape. The five hub genes include CD74, HLA-DOB, CIITA, STAP1 and CXorf21 (Fig. 8B). Then, we performed correlation analysis between these five hub genes and immune infiltration level for LUAD. Spearman’s correlation evaluated associations between gene and immune cells, and that associations were displayed in scatter plots. Hub genes were inversely proportional to cancer purity and proportional to immune cells (Fig. 8C).

### Development of a nomogram for OS of TCGA-LUAD

**Figure 9:**
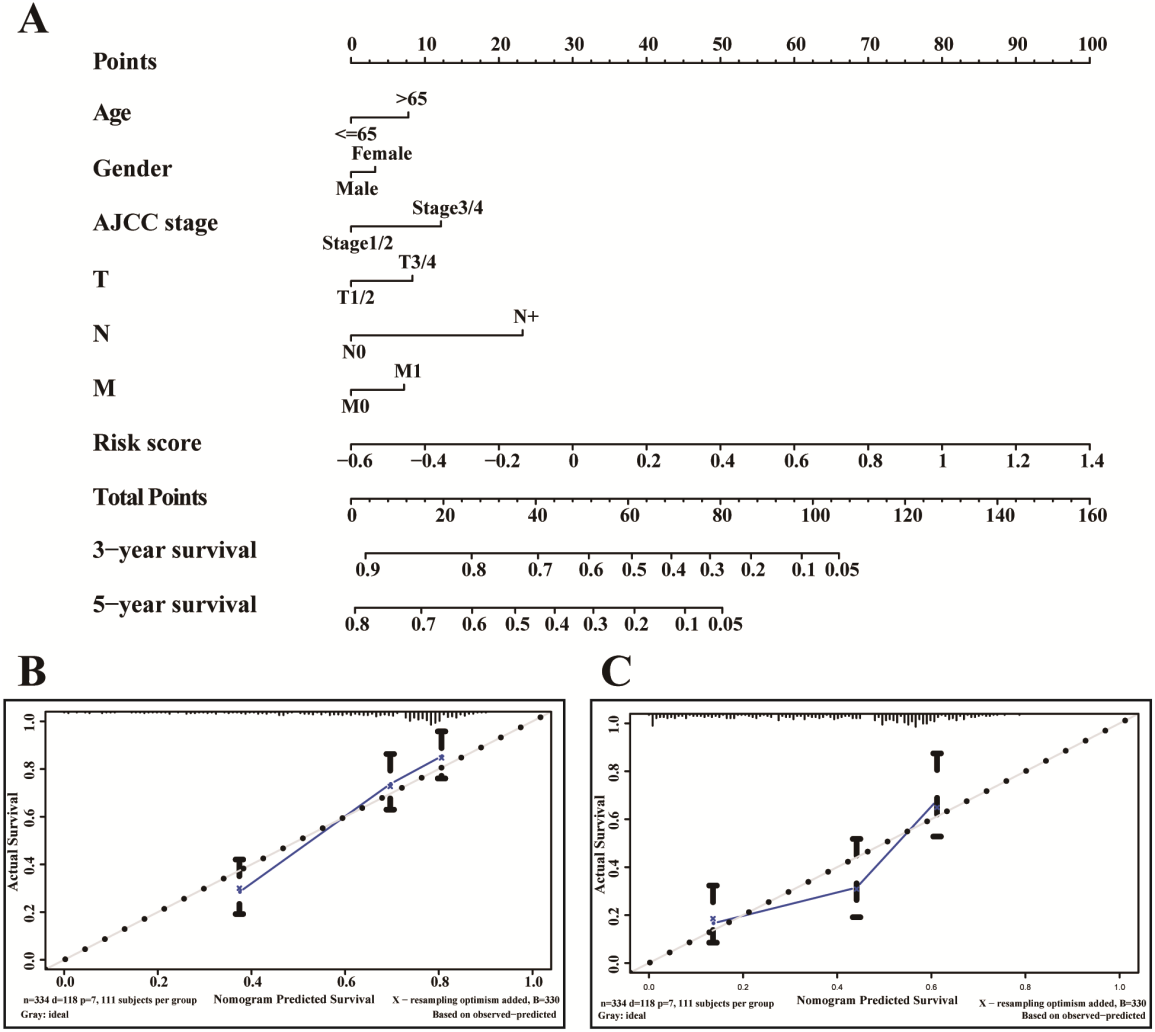
The nomogram of TCGA-LUAD. (A) The nomogram for predicting OS developed TCGA-LUAD cohort. (B) The calibration plots for predicting 3-year (C) and 5-year survival.

A nomogram is usually applied to quantitatively determine individuals’ risk in the clinical setting by integrating multiple factors. We designed a nomogram to predict the probability of 3- and 5-year OS of LUADs by synthesizing the gene signature, age, gender, T, N, M and AJCC stage (Fig. 9A). Each factor in the nomogram assigns points is in proportion to its risk contribution to survival. Calibration curves are often used to evaluate the accuracy of the model predictions, and our calibration curves indicated that actual and predicted survival were coincided very well especially for 3-year survival (Figs. 9B–9C).

## Discussion

In this work, we found that for stromal score, stage I and stage II patients are significantly higher than patients in stage IV (*P* < 0.05) (Fig. 2A), while for immune score, stage I patients are significantly higher than patients in stage III and stage IV (*P* < 0.05) (Fig. 2B), when analyzing the clinical data of LUAD from TCGA datasets. This result indicated that stromal and immune scores are related to the stage of LUAD. In addition, favorable OS was found in patients with high immune score (*P* = 0.028) (Fig. 2F), indicating that high immune score may show a good prognosis. After identification and selection of the immunological TME related DEGs and GO analysis, we constructed the prognosis model based on the immunological TME related DEGs. The Kaplan–Meier curve and log-rank test results showed that the low-risk group patients have significantly better OS (*P* = 2.501e−5 < 0.01) (Fig. 5C). To confirm the results in the training set, we validated the 397 LUAD patients with complete clinical information from GSE68465 and the result suggested a significant better overall survival in the low-risk group, which is consistent with the results in the training set (*P* = 2.443e−02 < 0.05) (Fig. 6C). Moreover, the multi-variable COX analysis demonstrated that only risk score was an important factor association with the OS of TCGA-LUAD (Fig. 7B), and risk score also showed significant association with the OS of GSE68465 cohorts (Fig. 7E). The AUC score of the risk score is 0.717 for the TCGA-LUAD and 0.632 for GSE68465, which also suggested that risk score can conduct as an independent prognostic factor of LUADs. In addition, we obtained the five most important genes (CD74, HLA-DOB, CIITA, STAP1 and CXorf21) as the five hub genes. In the immune infiltration analysis, the five genes are closely related with B cell, CD4+ T cell, CD8+ T cell, neutrophil, macrophage and dendritic cell infiltration (*P* < 0.05), suggesting a general rise in immune infiltration level. In addition, calibration curves showed that the predicted and actual survival matched well especially for 3-year survival (Figs. 9B–9C), which proved the validity and feasibility of the model. Moreover, in the result, the stromal score and immune score of samples in age >65 group are notably higher than samples in age ≤ 65 group (*P* < 0.05) (Figs. 2C–2D). The possible reasons of the stromal and immune score difference between >65 group and age ≤ 65 group could be as follows: Firstly, the correlation between immunity and age is a little controversial, with different results in different studies(Hirokawa et al., 2013; Tang et al., 2020; Weiss et al., 2016). Moreover, the original purpose of TCGA project is to design for molecular study. Therefore, sufficient tissue volume should be considered for genome research when samples are included, which will lead to certain preselection of TCGA patients and possibly lead to certain bias in some sample characteristics. Besides, the shortage of sample size may also be a part of the reason for this result. We consider that the larger size and more suitable samples may be needed to explore this result.

**Table 2:** The biological functions of the coding genes.

**Gene**	**Immune-related functions**
*CD70*	*CD70* belongs to TNF ligand family and can enhance the generation of cytolytic T cells and induce proliferation of co-stimulated T cells (Keller et al., 2007; Kuka et al., 2013). The regulatory mechanisms for the CD70-CD27 pathway is critical for prognosis of cancer patients.
*CXorf21*	*CXorf21* is a mediator of the X-chromosome gene of systemic lupus erythematosus in female (Bentham et al., 2015; Harris et al., 2019b) and is expressed only in immune cell (Harris et al., 2019a).
*CD74*	*CD74* expression serves as a prognostic factor in many cancers (Ekmekcioglu et al., 2016; Otterstrom et al., 2014; Ruvolo et al., 2019; Zeiner et al., 2015; Zeiner et al., 2018; Zhang et al., 2014).
*RUBCNL*	*RUBCNL* is a vertebrate-specific autophagy regulator (Cheng et al., 2017), and involves autophagy in NSCLC (Levy, Towers & Thorburn, 2017).
*BIRC3*	*BIRC3* involves in the prognostication system in Chronic Lymphocytic Leukemia (Alhourani et al., 2016) and hepatocellular carcinoma patients undergoing curative resection (Fu et al., 2019).
*TESC*	*TESC* can reinforce the radio-resistant properties of NSCLC (Lee et al., 2018).
*INSL4*	Abnormal *INSL4* signaling may act as a promising therapeutic target for LKB1-deficient NSCLC (Yang et al., 2018).
*HLA-DOB*	HLA-DOB are expressed in antigen presenting cells which is tightly linked with the immunity.
*CIITA*	The vaccination with CIITA-tumour cells is constructed to facilitate anti-tumour cells to enhance the immune system (Accolla et al., 2019).
*STAP1*	No relevant report yet.
*OBP2A*	No relevant report yet.

We studied the biological functions of the coding genes to improve our understanding of the underlying mechanisms of the immunological TME related carcinogenesis (Table 2). The protein encoded with gene CD70 is a cytokine, and it belongs to the tumour necrosis factor (TNF) ligand family which is viewed as a primary mediator of immune responses in the pulmonary environment. It can enhance the generation of cytolytic T cells and induce proliferation of co-stimulated T cells, which contributes to T cell activation (Keller et al., 2007; Kuka et al., 2013). Of note, the regulatory mechanisms for the CD70-CD27 pathway prevent deleterious immune responses (Kuka et al., 2013), which is critical for prognosis of cancer patients. Chromosome X open reading frame 21 (*CXorf21*) is a mediator of the X-chromosome gene dose-dependent increased risk of systemic lupus erythematosus in female (Bentham et al., 2015; Harris et al., 2019b) and is expressed only in immune cell (Harris et al., 2019a). The protein CD74 is expressed on antigen-presenting cells and reports showed that CD74 expression serves as a prognostic factor in many cancers (Ekmekcioglu et al., 2016; Otterstrom et al., 2014; Ruvolo et al., 2019; Zeiner et al., 2015; Zeiner et al., 2018; Zhang et al., 2014). RUBCNL, also known as PACER, is identified as a vertebrate-specific autophagy regulator (Cheng et al., 2017), and studies have showed the implication of autophagy in NSCLC (Levy, Towers & Thorburn, 2017). In addition, BIRC3 is reported to improve the prognostication system in Chronic Lymphocytic Leukemia (Alhourani et al., 2016) and hepatocellular carcinoma patients undergoing curative resection (Fu et al., 2019). It was reported that TESC can reinforce the radio-resistant properties of NSCLC (Lee et al., 2018), which is critical for prognosis. HLA-DOB, which is anchored in the membrane, are expressed in antigen presenting cells. A research reported that aberrant INSL4 signaling may act as a promising therapeutic target for LKB1-deficient NSCLC (Yang et al., 2018). Accolla et al., (2019) constructed the vaccination with CIITA-tumour cells to facilitate the triggering and persistence of anti-tumour cells to enhance the immune system. Although STAP1 and OBP2A remain inadequately investigation in TME or cancer related research, our results might provide some clues for further studies.

In recent years, more and more machine learning methods have also been applied to the construction of prognosis models, which may provide valuable reference for clinical decision-making. For example, Li et al., (2019) also used LASSO regression analysis to developed a 16-gene-based risk model for LUAD prognosis prediction. But the difference is that, the genes we screened are about TME of LUAD and our 11-gene-based prognostic model is essentially a bridge between TME and OS of LUAD patients, which may be an innovation point of our study.

## Conclusions

To sum up, we constructed and confirmed an 11-gene signature-based risk score model which can perform as an independent prognostic factor of LUADs especially for 3-year survival. We also demonstrated that LUAD patients with high infiltration of immune and stromal cells may express better prognosis. The result of this work shows that the 11-gene signature risk score model may help to facilitate personalized medicine in the NSCLC treatment.

##  Supplemental Information

10.7717/peerj.10749/supp-1 Supplemental Information 1 TCGA-LUAD samples with survival information and ESTIMATE scores The raw data including TCGA ID, overall survival time, survival status and ESTIMATE scores.

10.7717/peerj.10749/supp-2 Supplemental Information 2 High- and low- immune score group list of TCGA-LUAD The first 239 samples are high immune score group, and the last 238 samples are low immune score group.

10.7717/peerj.10749/supp-3 Supplemental Information 3 The GO enrichment results of DEGs. (Top 10 terms of BP, MF and CC) The GO enrichment category, GO-ID, GO-term, genes and adjusted P value.

10.7717/peerj.10749/supp-4 Supplemental Information 4 Symbol id and logFC values of DEGs Gene symbols and logFC values of all DEGs.

10.7717/peerj.10749/supp-5 Supplemental Information 5 TCGA-LUAD samples with survival information and DEGs, which could be matched in GSE68465 dataset

10.7717/peerj.10749/supp-6 Supplemental Information 6 Genes filtered by univariate COX regression analysis After univariate COX regression analysis, genes with P¡0.05 were filtered.

10.7717/peerj.10749/supp-7 Supplemental Information 7 GSE68465 samples with survival information and genes filtered by above univariate COX regression analysis

10.7717/peerj.10749/supp-8 Supplemental Information 8 TCGA-LUAD samples with survival information, genes filtered by LASSO analysis and risk score

10.7717/peerj.10749/supp-9 Supplemental Information 9 GSE68465 samples with survival information, genes filtered by LASSO analysis and risk score

10.7717/peerj.10749/supp-10 Supplemental Information 10 Clinical features of TCGA-LUAD samples Clinical features including sample ID, age, gender, AJCC stage, and TNM stage.

10.7717/peerj.10749/supp-11 Supplemental Information 11 Clinical features of GSE68465 samples Clinical features including sample ID, age, gender, and TN stage.

10.7717/peerj.10749/supp-12 Supplemental Information 12 Clinical information of TCGA-LUAD for nomogram Clinical information of TCGA-LUAD for nomogram including sample ID, age, gender, AJCC stage, and TNM stage.

10.7717/peerj.10749/supp-13 Supplemental Information 13 Raw code R

## Additional Information and Declarations

### Data Availability

The following information was supplied regarding data availability: The raw measurements are available in the Supplemental Files. TCGA-LUAD mRNA FPKM data is available at Figshare: Hao, Lizheng (2021): dat2.zip. figshare. Dataset. https://doi.org/10.6084/m9.figshare.13515998.v1.
